# Supplementary material for: The acquisition of rmpADC can increase virulence of classical Klebsiella pneumoniae in the absence of other hypervirulence-associated genes
Source: mBio. 2025 Dec 9;17(1):e03122-25. doi: 10.1128/mbio.03122-25 (PMC12802315; doi:10.1128/mbio.03122-25)
Supplement: Supplemental Figures — Figures S1 to S5. [file mbio.03122-25-s0001.pdf]

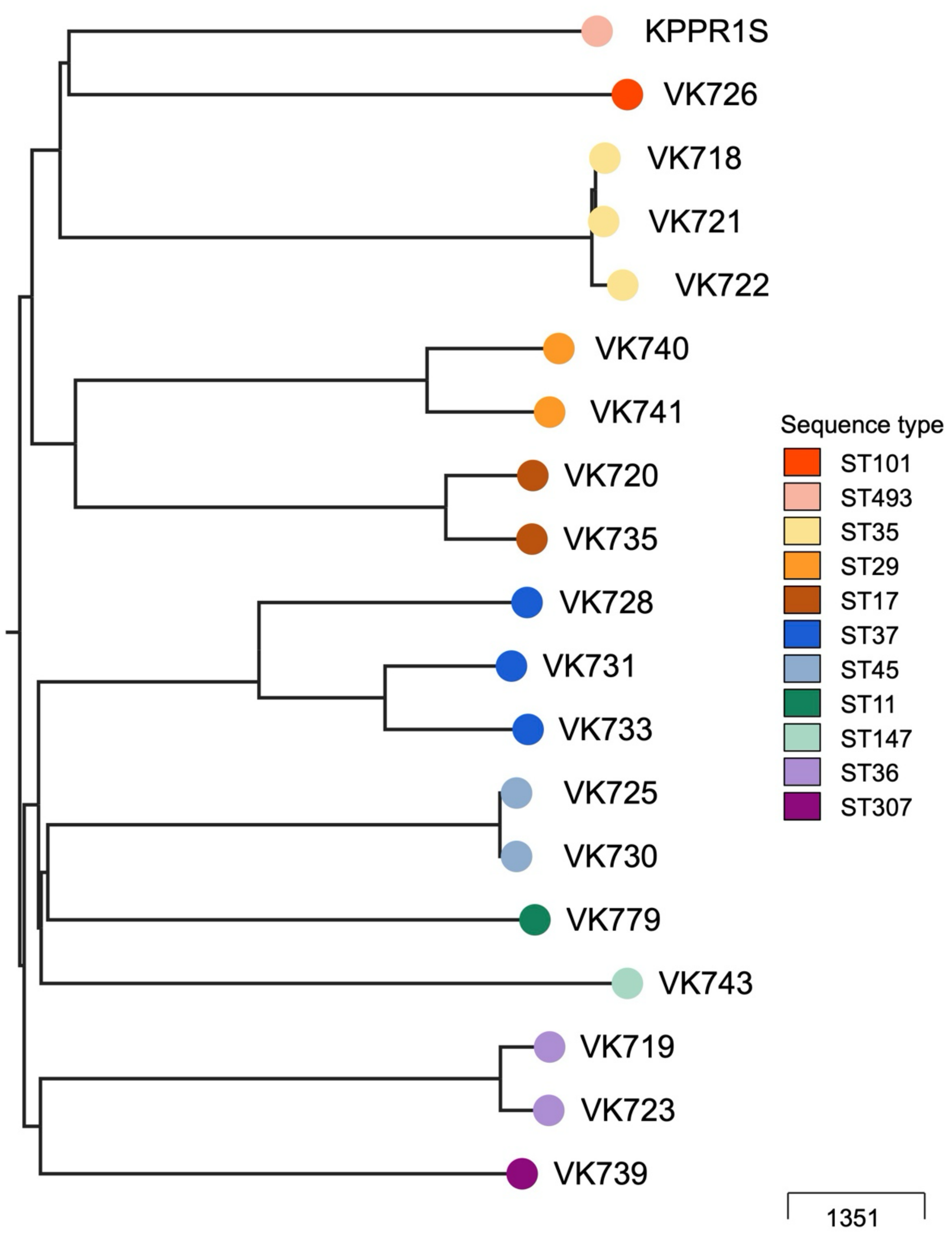

Figure S1. Phylogenetic relationships of the strains used in this study. Genome sequences were aligned against the *K. pneumoniae* core library using Pathogenwatch. The colored dots indicate different sequence types (ST), as indicated.

Fig. S2, page 1 of 3

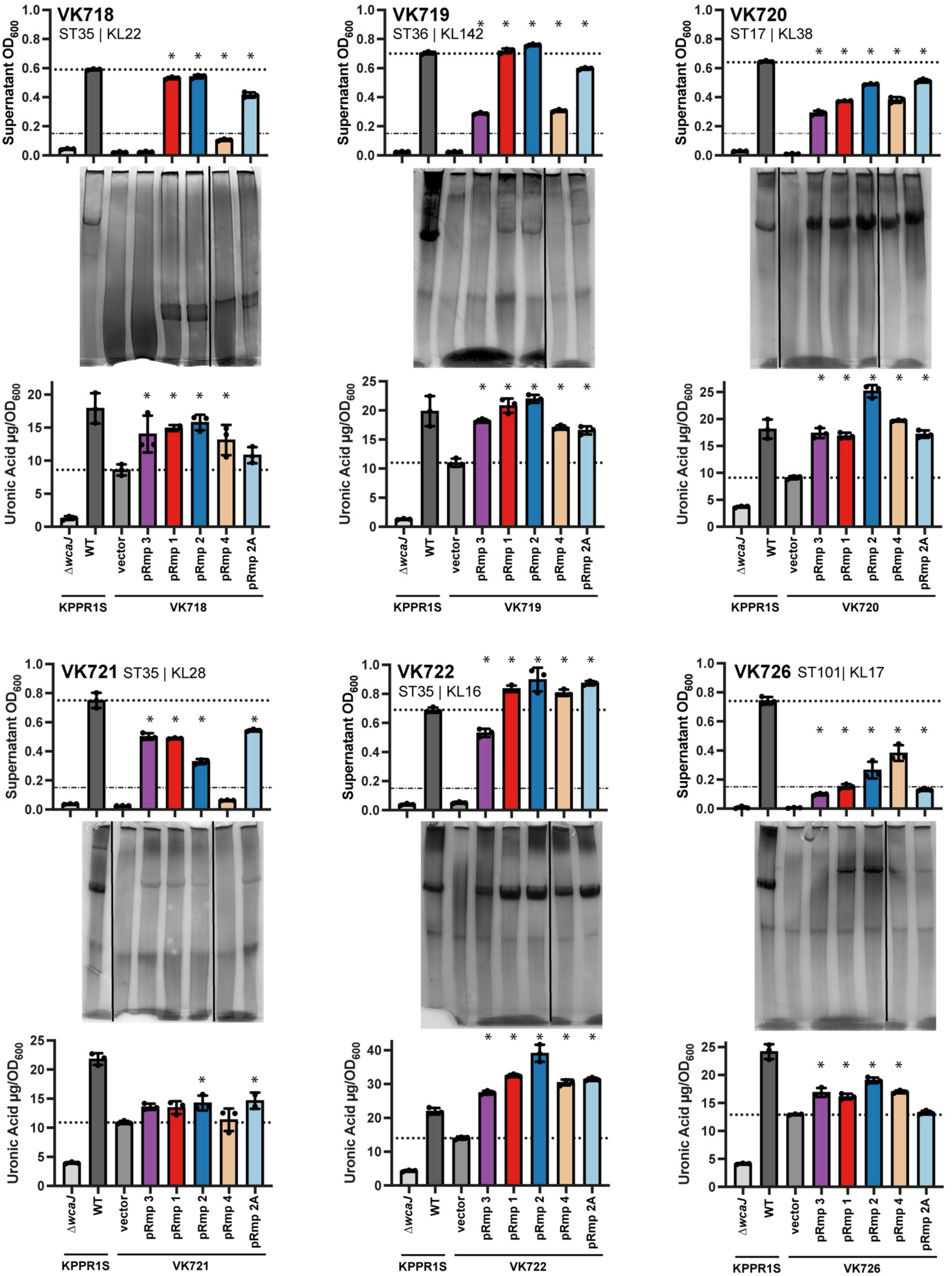

Fig. S2, page 2 of 3

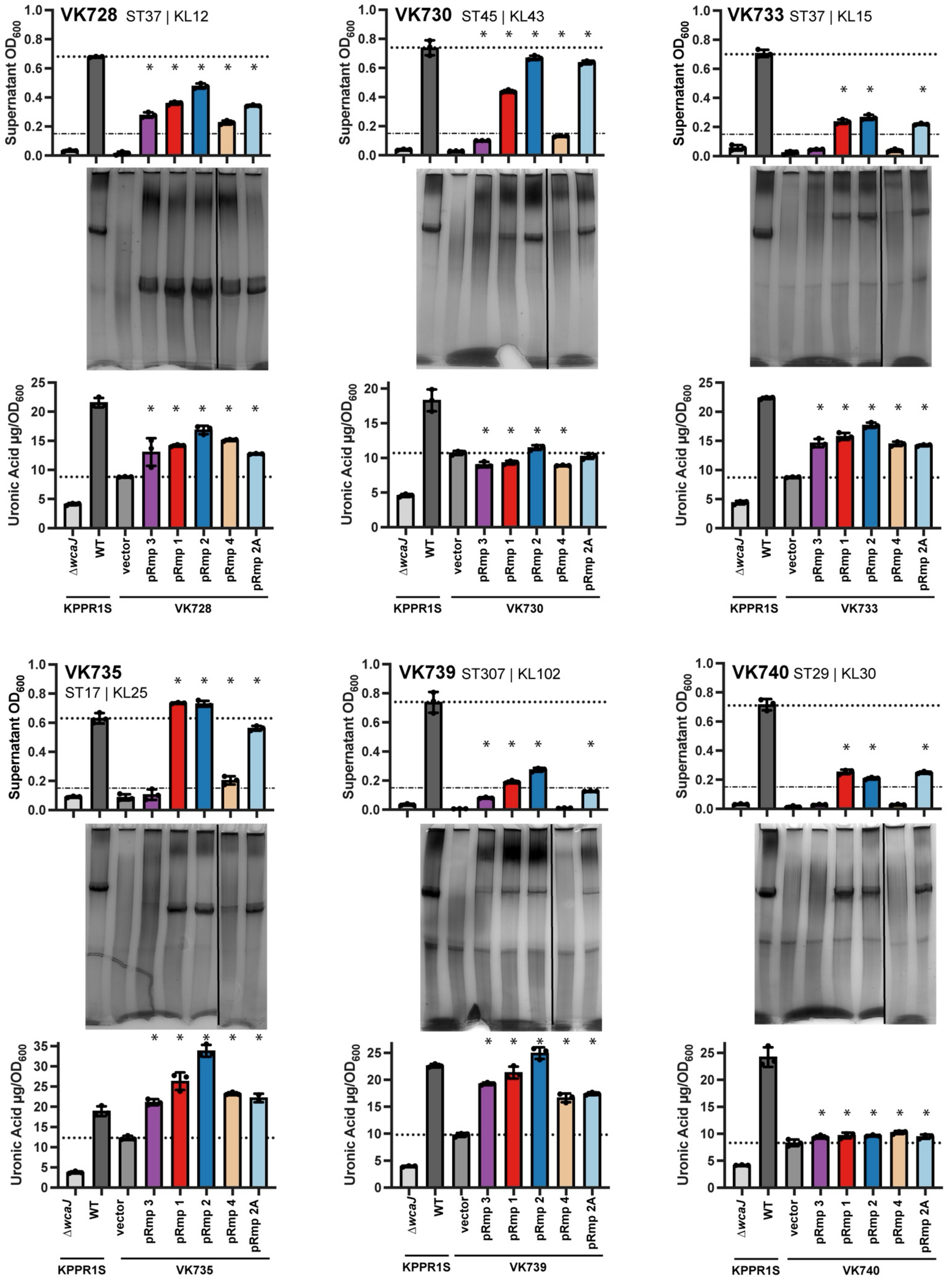

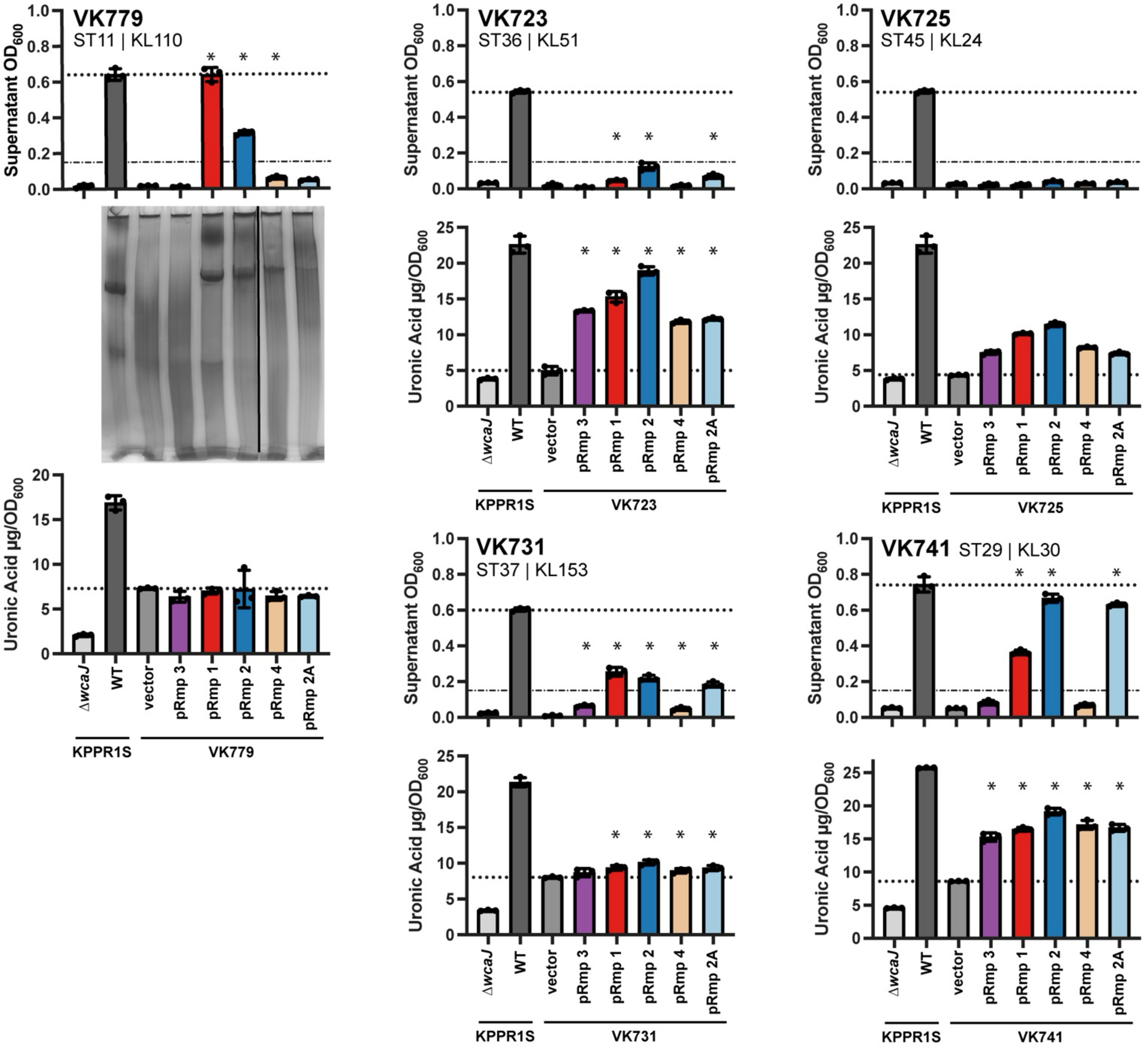

Figure S2. Graphical representation of the mucoviscosity and capsule production data in Fig. 1 and corresponding capsule gel electrophoresis from cKp with plasmid-borne *rmpADC*. For each strain, the top graph is sedimentation resistance (mucoviscosity) and the bottom graph is uronic acid quantification (proxy for capsule amount). In the mucoviscosity graphs, the dotted (…) line is set at the level of the KPPR1S control. The dot-dash-dot (._.) line is set to 0.15, a threshold value for determining if a strain is HMV+ or HMV- (40). In the UA graphs, the dotted (…) line is set at the level of the parental strain with the vector control to better visualize increases in strains expressing the *rmp* genes. Δ*wcaJ* is a capsule deficient strain used as a negative control. One-way ANOVA with Dunnett's post-test compared to each parental strain was used to determine statistical significance. Some had very low *P*-values, but for simplicity, * indicates *P* < 0.05. The middle images show capsule gel electrophoresis of corresponding samples from the graphs (not conducted for all strains). Proteinase K-treated whole cell lysates were run, then stained with alcian blue and silver to visualize capsule chain patterns. Strains that are HMV- have a smear of variable capsule chain lengths. HMV+ strains produce a defined band of uniform length and sometimes a slight smear of high molecular weight material.

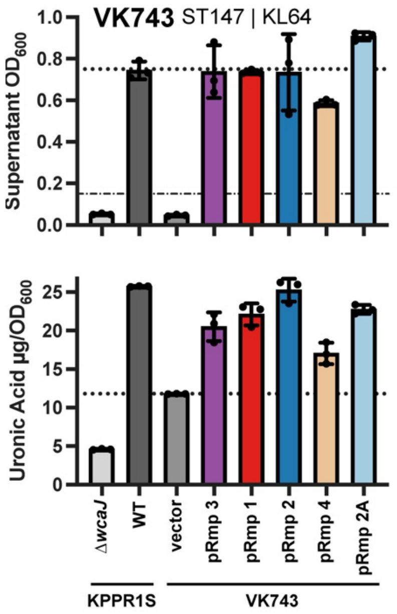

A. Pearson's correlation analysis of data in Fig. 1

Pearson's r

	HMV-1	HMV-2	HMV-2A	HMV-3	HMV-4	UA-1	UA-2	UA-2A	UA-3	UA-4
HMV-1	1.00	0.87	0.78	0.66	0.61	-0.13	-0.14	0.05	-0.13	-0.14
HMV-2	0.87	1.00	0.92	0.61	0.68	0.01	0.05	0.23	0.04	0.08
HMV-2A	0.78	0.92	1.00	0.77	0.69	0.03	0.05	0.32	0.09	0.07
HMV-3	0.66	0.61	0.77	1.00	0.69	0.07	0.05	0.32	0.14	0.00
HMV-4	0.61	0.68	0.69	0.69	1.00	0.16	0.22	0.34	0.20	0.26
UA-1	-0.13	0.01	0.03	0.07	0.16	1.00	0.97	0.92	0.96	0.89
UA-2	-0.14	0.05	0.05	0.05	0.22	0.97	1.00	0.92	0.96	0.94
UA-2A	0.05	0.23	0.32	0.32	0.34	0.92	0.92	1.00	0.94	0.87
UA-3	-0.13	0.04	0.09	0.14	0.20	0.96	0.96	0.94	1.00	0.90
UA-4	-0.14	0.08	0.07	0.00	0.26	0.89	0.94	0.87	0.90	1.00

P values

	HMV-1	HMV-2	HMV-2A	HMV-3	HMV-4	UA-1	UA-2	UA-2A	UA-3	UA-4
HMV-1		1.4E-06	8.6E-05	2.1E-03	5.3E-03	0.59	0.58	0.83	0.60	0.56
HMV-2	1.4E-06		2.9E-08	5.6E-03	1.5E-03	0.95	0.85	0.35	0.86	0.75
HMV-2A	8.6E-05	2.9E-08		9.9E-05	1.1E-03	0.90	0.84	0.19	0.72	0.78
HMV-3	2.1E-03	5.6E-03	9.9E-05		1.0E-03	0.78	0.83	0.18	0.56	1.00
HMV-4	5.3E-03	1.5E-03	1.1E-03	1.0E-03		0.50	0.37	0.15	0.42	0.29
UA-1	0.59	0.95	0.90	0.78	0.50		7.8E-12	2.1E-08	2.6E-11	2.5E-07
UA-2	0.58	0.85	0.84	0.83	0.37	7.8E-12		3.5E-08	3.9E-11	3.7E-09
UA-2A	0.83	0.35	0.19	0.18	0.15	2.1E-08	3.5E-08		1.2E-09	9.7E-07
UA-3	0.60	0.86	0.72	0.56	0.42	2.6E-11	3.9E-11	1.2E-09		1.1E-07
UA-4	0.56	0.75	0.78	1.00	0.29	2.5E-07	3.7E-09	9.7E-07	1.1E-07	

B. PCA plot of data in Fig. 1

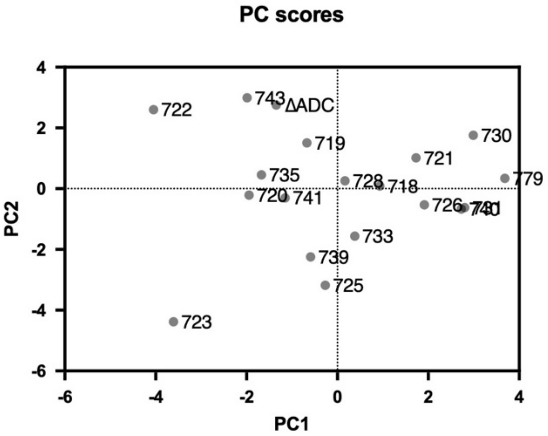

Figure S3. Correlation and principal component analysis of HMV and UA data from Fig. 1 and S2. A. Two-tailed Pearson's correlation coefficient calculations were performed with GraphPad Prism, v10. Top panel has the correlation values (Pearson's r) and the bottom panel has the corresponding *P* values. B. PCA plot showing the relationships of the HMV and UA data (generated by GraphPad Prism, v10).

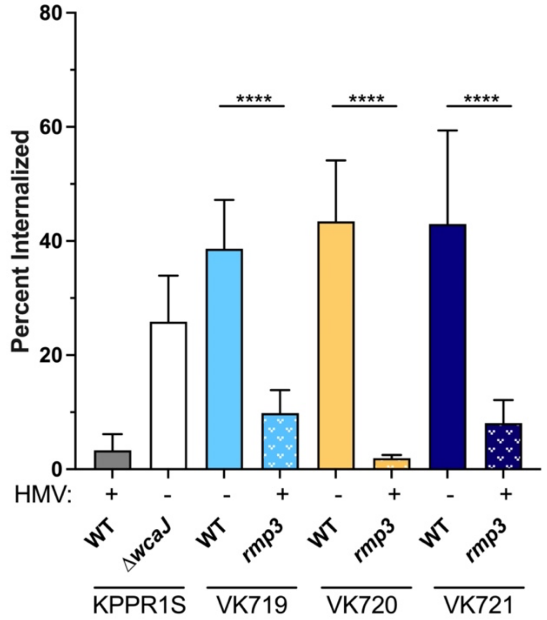

Figure S4. *rmp* acquisition blocks phagocytosis in an HMV-dependent manner.
Internalized bacteria was determined as described in Materials and Methods. Data were obtained from three biological replicates. Bars represent average values while error bars indicate standard deviations. One-way ANOVA with Tukey's post-test was used to determine significance compared to parental strains. ns, not significant; ***, $P < 0.001$; ****, $P < 0.0001$.

Lung strain	Sample	*rmpA*	*gyrB*
VK719*rmp3*	A	22.57	28.82
	B	23.51	31.23
	C	25.51	34.15
VK720*rmp3*	A	34.17	37.93
	B	33.67	38.44
	C	32.02	NaN
VK721*rmp3*	A	33.40	NaN
	B	35.04	38.10
	C	32.61	NaN

Figure S5. *rmpA* is expressed *in vivo*. Average C_T values from qRT-PCR analysis of RNA isolated from the lungs of three mice infected with indicated *K. pneumoniae* strains. *gyrB* was not detected in all samples of VK720*rmp3* and VK721*rmp3* infected tissues, possibly due to the lower bacterial burden in those tissues.
