# Supplementary material for: The acquisition of rmpADC can increase virulence of classical Klebsiella pneumoniae in the absence of other hypervirulence-associated genes
Source: mBio. 2025 Dec 9;17(1):e03122-25. doi: 10.1128/mbio.03122-25 (PMC12802315; doi:10.1128/mbio.03122-25)
Supplement: Table S1 — Primers used during this study. [file mbio.03122-25-s0002.docx]

**Table S1. Primers used in this study.**

| **Primer #** | **Sequence 5 - 3** | **Use** | **Reference** |
| --- | --- | --- | --- |
| SMS015 | CATCGCCTTCTATCGCCTTCTTG | pEW103 flank | This work |
| SMS016 | GGATTATTTAGGGAAGAGTGAC | pEW103 flank | This work |
| SMS031 | TTGGCCTGCAAGGCCTCCTTTGTTGAACAATTCCATG | amplify *rmp3* | This work |
| SMS034 | GTTTTGCTGCCCTCGAGGGACACCAAAAGTTATACCATC | amplify *rmp3* | This work |
| SMS046 | TTGGCCTGCAAGGCCTCTTTGTTGAACAATTCCATGC | amplify *rmp1* | This work |
| SMS047 | GTTTTGCTGCCCTCGAGGGAAACAAAAAGCTATACCATC | amplify *rmp1* | This work |
| KW370 | AGTTAACTGGACTACCTCTGTTTC | *rmpA*-Fwd qRT-PCR | This work |
| KW371 | TCCTGCAGTCAACCAATACTC | *rmpA*-Rev qRT-PCR | This work |
| *gyrB*-Fwd | CCGAGCTGAACGAGAAAGAA | *gyrB*-Fwd qRT-PCR | This work |
| *gyrB*-Rev | GTGGGTACGTACGCGAATAA | *gyrB*-Rev qRT-PCR | This work |
